# Effects of Integrated Health Management Intervention on Overweight and Obesity

**DOI:** 10.1155/2017/1239404

**Published:** 2017-01-01

**Authors:** Yiting Yang, Chung Wah Ma, Yide Yang, Xiaoling Wang, Xiaoliang Lin, Lianguo Fu, Shuo Wang, Zhongping Yang, Zhenghe Wang, Xiangkun Meng, Dongmei Ma, Rui Ma, Jun Ma

**Affiliations:** ^1^Research & Development Centre, Infinitus (China) Company Ltd., Guangzhou, China; ^2^Institute of Child and Adolescent Health, School of Public Health, Peking University Health Science Center, Beijing, China; ^3^Department of Preventive Medicine, Bengbu Medical College, Bengbu City, Anhui, China

## Abstract

Overweight or obese adults aged 20~55 years and living in Beijing more than one year were randomly divided into different management groups. A one-year integrated health management intervention was applied in the health management groups. The physical indicators and metabolic indicators changed after one-year intervention on the overweight and obese adults. The annual reduction of the physical indicators was significant in all groups (*p* < 0.05) except the weight loss in the placebo + general management group. The health management and the dietary supplement have statistically significant (*p* < 0.001, *p* < 0.001) effects on the annual reduction of these indicators and interactive effect between them was found on some of these indicators such as bodyweight, body mass index (BMI), body fat ratio (BFR), and hipline (*p* < 0.05). The dietary supplement + health management group had the best annual reduction effects for the indicators among the groups. Integrated health management interventions including both dietary supplements intervention and health management could improve metabolic indicators in overweight and obese adults together with the physical indicators, suggesting the intermediated role of metabolic indictors in controlling obesity.

## 1. Introduction

Overweight/obesity is epidemic worldwide and also in China with the number of obese patients increasing at a significant rate each year [1, 2]. The escalating rates of obesity parallel the increases in the number of chronic diseases such as cardiovascular disease, diabetes, and hypertension [3]. With the advances in the research of obesity and the related chronic diseases over the years, some metabolic indictors were found to correlate with these diseases [4]. For example, there is evidence that high serum C-reactive protein (CRP) is related to overweight or obese status and also involved in the development of several chronic diseases such as obesity, cardiovascular diseases, type 2 diabetes mellitus, and colorectal and other cancers [5–7]. The role of diet or exercises in reducing serum CRP and thereby modulating obesity was also supported by large body of evidences [8–10]. Therefore, changes in these metabolic indicators might affect the outcomes of overweight/obesity intervention. To date, no study has investigated the effect of integrated health management interventions including both dietary supplements intervention and health management on these metabolic indicators. Therefore, in this study an integrated health management intervention was applied and the effectiveness of intervention was evaluated by physical examinations and by measurement of different metabolic indicators.

## 2. Subjects and Methods

### 2.1. Subjects

Subjects were recruited voluntarily through a recruitment notice and BMI is used as the screening indicator. Subjects who met the following criteria were recruited: (1) males and females aged 20–55 years; (2) having lived in their cities for at least one year; (3) meeting the diagnostic criteria in the Guidelines for the Prevention and Control of Overweight and Obesity in Chinese Adults (2006): overweight: 24 kg/m^2^ ≤ BMI < 28 kg/m^2^; obesity: BMI ≥ 28 kg/m^2^. The exclusion criteria were as follows: (1) suffering from certain severe organic diseases; (2) secondary obesity arising from illness; (3) women with pregnancy plan, pregnant women, and lactating women; (4) taking any other constitutional conditioning or weight loss product before or during intervention.

### 2.2. Intervention Program

The intervention period was one year. Intervention measures include health management, general management, dietary supplements, and placebo. (1) Health management: health management includes nutrition, exercise, and behavioral correction. Methods such as health education, regular health checkup, telephone interview, and behavioral diary were used for integrated management and guidance. A novel “3-2-1-0” behavioral plan including main dietary and exercise actions was also set to improve the compliance of subjects and monitor the health management intervention. In this plan, “3” represented eating vegetables of at least 3 fists' size per day; “2” represented eating meat of 2 palms' size per day; “1” represented walking 10,000 steps per day; and “0” represented not eating high-sugar snacks. (2) General management: general management was performed under the same physical monitoring as the health management groups without special management, offering multiple physical checkup reports. (3) Dietary supplements: the dietary supplements used in this study were extracted and refined from edible materials, such as poria and mushroom, and added with edible seasonings. In addition, the dietary supplements had constitutional conditioning effects according to Traditional Chinese Medicine. The slimming tablets could invigorate the spleen and induce diuresis, dietary fiber could supplement dietary fiber to increase satiation, Gest-Aid Plus could regulate the stomach and spleen, Yi Rui Capsule could activate blood circulation to remove stasis, and Schizandra Plus could supplement Qi. They usually have no adverse reaction and had been certified by the State Food and Drug Administration for safety and efficacy through human and animal experiments. The dietary supplements were administrated twice a day, at lunch and supper. (4) The placebo was a preparation free from any pharmacological ingredient, prepared from edible materials, similar to the foods and health products in shape. The placebo was administrated twice a day at lunch and supper.

### 2.3. Intervention Grouping

**Figure 1:**
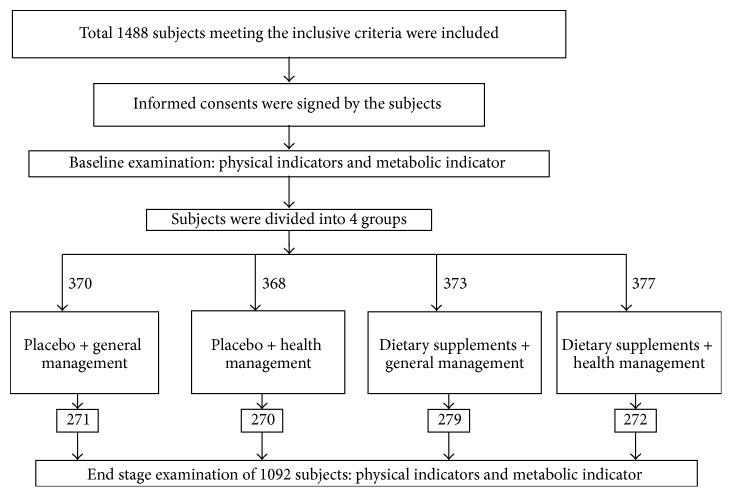
Schematic picture of the procedure of the one-year intervention.

Subjects were divided into 4 groups by simple randomization according to the type of intervention measurements. Group 1: placebo + general management (P + GM); Group 2: placebo + health management (P + HM); Group 3: dietary supplements + general management (DS + GM); Group 4: dietary supplements + health management (DS + HM). The dietary and placebo studies were double-blinded. Neither subjects nor the medical staffs were aware of the grouping situation. Study procedures and grouping were listed in Figure 1 and loss of samples at different stages was shown in Table 1.

### 2.4. Measuring Indicators

The physical indicators were measured through health checkup at baseline, month 1, month 3, month 6, month 9, and month 12 of intervention, respectively. The indicators included height, bodyweight, body fat ratio (BFR), waistline, and hipline. Mean value was taken after measurement. Height was measured twice at each time point in cm and accurate to 0.1 cm, and if error exceeded 0.5 cm, height would be measured three times. Each subject stands upright on the base of the height meter with bare feet, no hat, and close-fitting underwear. Weight was measured twice in kg and accurate to 0.1 kg. If error exceeds 0.1 kg, it would be measured three times. Waistline and hipline were measured twice in cm and accurate to 0.1 cm. They would be measured three times if error exceeds 1.0 cm. BFR was checked by TANITA, MC-180 body composition analyzer, for any metallic implant, such as tooth brace, pacemaker, and joint and accurate to one decimal place. The 14 metabolic indicators measured at baseline, middle stage (month 6), and final stage (month 12) include high-density lipoprotein cholesterol (HDL), low-density lipoprotein cholesterol (LDL), triglyceride (TG) and total cholesterol (TC), leptin (LEP), adiponectin, fasting insulin level (INS), fasting blood sugar (GLU), free fatty acids (FFA), glycosylated hemoglobin (HbA1C), insulin resistance index (HOMAIR), insulin sensitivity index (HOMAIS), tumor necrosis factor *α* (TNF-*α*), and C-reactive protein (CRP). Calculation method for key insulin-related derivative indicators (steady-state model evaluation): insulin resistance index (HOMAIR) = fasting plasma glucose (GLU, mmol/L) × fasting insulin (INS, mIU/L)/22.5; insulin sensitivity index (HOMAIS) = 1/[(fasting plasma glucose (GLU, mmol/L) × fasting insulin (INS, mIU/L)] [11].

### 2.5. Ethical Application and RCT Registration

This project has been approved by the Ethical Committee of Peking University Health Science Center. The progress report has been submitted and approved, and the ethical closing application is ongoing. The number of the ethical certificate is IRB00001052-13086. This project has been registered on ClinicalTrials.gov, with RCT ID of NCT02298426.

### 2.6. Statistical Analysis Methods

The SPSS20.0 software is used for a statistical analysis. Measured data is expressed in mean ± standard deviation. The statistical methods include factorial design variance analysis, single-factor variance analysis, multiple logistic regression analysis, and chi-square test, where *p* < 0.05 denotes a statistically significant difference. Data is analyzed using the Per-Protocol (PP) analysis method, and the analysis is for subjects complying with intervention only.

## 3. Results

### 3.1. Physical Indicators Changings

Total 1488 subjects were recruited to this study and 1092 completed. The other subjects were excluded because of the following reasons: (1) long bone density measuring time or perceived irradiation; (2) not attending physical checkup (busy working, trip, etc.); (3) poor compliance (doubting product safety, etc.); (4) loss of contact (not answering calls, shutdown, etc.); (5) physical conditions (pregnancy/readiness for pregnancy, etc.); (6) sickness of self/family member. All the 1,092 subjects in this study that completed one-year health interventions had no statistically significant difference in age, gender, and baseline BMI among the four intervention groups (data were not shown). After this one-year intervention on the overweight and obese adults, the annual reduction of the physical indicators (Table 2) such as bodyweight, BMI, BFR, hipline, and waistline was significant in all four groups (*p* < 0.05) except the weight loss (−0.23 kg) in the placebo + general management group. It was also found that both the health management and the dietary supplement had statistically significant (*p* < 0.001, *p* < 0.001) effects on the annual reduction of these indicators. In addition, there was interactive effect between the health management and the dietary supplement in annual reduction of some of the key indicators such as bodyweight, body mass index (BMI), body fat ratio (BFR), and hipline (*p* < 0.05). Based on pairwise comparison, the differences between any two of the four groups are statistically significant for these indicators (*p* < 0.05). The dietary supplement + health management group had the best annual reduction effects for the indicators among the four groups as shown in Table 2 with annual weight loss of 4.24 kg, annual BMI loss of 1.60 kg/m^2^, annual WC loss of 6.61 cm, and annual hipline loss of 3.25 cm. The effective rates of weight loss of the four groups are 3.30%, 31.11%, 34.78%, and 42.86%, respectively, with the dietary supplements + health management having the highest effective rate of annual weight loss of 5%. Based on chi-square test, the differences among the four groups are statistically significant as in Table 2 (*χ* ^2^ = 40.02, *p* < 0.001).

### 3.2. Metabolic Indicators Changings

Following the improvement of the physical indicators, the metabolic indicators had also changed after the one-year intervention. It was found from the pairwise comparison of metabolic indicators at baseline and month 12 among the four groups (Table 3) that there was no statistically significant difference among the four groups. However, there were declines of hypersensitive C-reactive protein (CRP), total cholesterol (TC), triglyceride (TG), free fatty acids (FFA), blood sugar (GLU), insulin (INS), HOMA-Insulin Resistance (HOMAIR), leptin (LEP), HbA1C, low-density lipoprotein (LDL), and tumor necrosis factor (TNF) and increased level of high-density lipoprotein (HDL) and HOMA-Insulin Sensitivity (HOMAIS). From the analysis, the annual declines in C-reactive protein, leptin, triglyceride, blood sugar, and HOMAIR were the best in the dietary supplements + health management group while HDL was higher in the dietary supplements + health management group. In order to explore effects of the dietary supplements and health management program on the metabolic indicators in overweight/obese adults and their interaction, a linear mixed model was built at baseline, month 6, and month 12 with/without dietary supplements intervention and with/without health management, and the results were shown in Table 4. Effects of with/without dietary supplements intervention on the reduction of CRP, leptin, insulin, TC, LDL, blood sugar, FFA, HOMAIR, and HOMAIS were statistically significant (*p* < 0.05), while the effects of with/without health management on the reduction of leptin, insulin, TG, HOMAIR, and HOMAIS and on the rise of HDL were statistically significant (*p* < 0.05). The interactions between dietary supplements intervention and health management were statistically insignificant (*p* > 0.05) for all the indicators.

### 3.3. Correlation Analysis

Based on the correlation analysis between weight loss and metabolic changes, the annual declines in C-reactive protein, insulin, total cholesterol (TC), LDL, leptin, triglyceride (TG), blood sugar (GLU), and HOMAIR were positively correlated with weight loss, and the correlation coefficients are statistically significant (Table 5); the annual rises in HOMAIS were positively correlated with weight loss, and the correlation coefficient is statistically significant (*p* < 0.001) (Table 5).

## 4. Discussions

Obesity is a severe problem worldwide currently. Chronic diseases such as hypertension, cardiovascular event, and type II diabetes mellitus are often related to obesity. Weight loss results in drop of blood pressure, improvement of lipid, and decrease of the incidence of diabetes mellitus. Diet, exercise, and behavioral correction are three effective ways that are being used to control overweight and obesity. However, there was no comprehensive study of integrated health management intervention including both dietary supplement and health management on overweight and obesity previously. In this study, the effects of integrated health management intervention including dietary supplements and health management intervention on obese/overweight adults were investigated. The effects were measured by physical indicators changes such as decline of bodyweight, BMI, BFR, waistline, and hipline and the effective weight loss of 5% in overweight and obese adults. The correlation of dietary supplements and health management intervention on each indicator was studied and interactive effect between them was also studied. Our data showed that integrated health management intervention had the best results on reduction of these key physical indicators. Comparing with other groups, integrated health management intervention showed significant difference, suggesting that combination of dietary supplements and health management intervention was better than dietary supplements or health management intervention alone. These data were consistent with our previous study, in that we also showed the interactive effect between dietary supplements and health management intervention on obese people based on constitutional classification [12].

In addition to observation of the improvement of physical indicators, changes of the metabolic indicators were also studied to find the underlying mechanism of integrated health management intervention on overweight and obesity. Based on the correlation analysis between weight loss and metabolic changes, the annual declines in CRP, insulin, triglyceride, blood sugar, and insulin resistance index were positively correlated with weight loss, and the correlation coefficients were statistically significant. The annual rises in insulin sensitivity index were positively correlated with weight loss, and the correlation coefficient was also statistically significant. These results suggested the possibility of integrated health management intervention, including diet, exercise, and behavioral correction, controlling overweight and obesity by improving different metabolic indicators. In previous studies, weight loss, exercise, and smoking all decreased CRP level [13–15]. Association between aerobic exercise and low levels of high sensitivity-CRP (hs-CRP) was previously observed [16, 17]. Previous studies also showed that CRP level was related to dietary factors. High intakes of vegetables and fruit were associated with lower levels of circulating hs-CRP [18]. It might be used to explain the lower serum CRP levels in Asian population than the Western populations besides genetic factors [19]. Meta-analysis of randomized controlled trials also suggested dietary factors in regulating serum level of CRP. In obese populations, flaxseed and its derivatives may significantly reduce CRP [16]. Carotenoids and vitamin C and vitamin D supplementation significantly decreased the circulating hs-CRP level as shown by meta-analysis [20]. A significant reduction in CRP levels was also seen in vitamin E-treated individuals [21]. In this study, dietary supplements administrated were containing fibers and other special supplements that could regulate constitution according to Chinese Medicine. They might control obesity by improving metabolic indicators. In fact, CRP level significantly decreased after different interventions in all four groups, suggesting both dietary supplements and health managements had effect on CRP decline. In addition, the annual decline in CRP was best in the dietary supplements + health management group, suggesting that intergraded health management had better effect on the annual decline of CRP. However, data analysis showed that dietary supplements intervention on the reduction of CRP was statistically significant while health management on the reduction of CRP was statistically insignificant and the interaction between dietary supplements intervention and health management was statistically insignificant. These results suggested that CRP decline in this one-year study was more related to the dietary factors other than health management. The same results were seen in blood glucose, LDL, and FFA. From the analysis of other metabolic indicators such as leptin, insulin, HOMAIR, HOMAIS, and TG, both dietary supplements intervention and health management intervention on the reduction of these indicators were statistically significant. These results supported that integrated health management could control overweight and obesity by improving different metabolic indicators through either dietary supplements intervention or health management intervention or both.

Moreover, all the metabolic indicators including TC, TG, FFA, GLU, insulin, HOMAIR, HOMAIS, leptin, HbA1C, HDL, LDL, and TNF were previously proved to have the relationship with chronic disease of hypertension, diabetes mellitus, and cardiovascular disease [22, 23]. CRP has also been intensively studied and meta-analysis for the relationship of CRP with hypertension, cardiovascular disease, and diabetes mellitus suggested its role in mediating these chronic diseases [24, 25]. Therefore, the parallel changes of these metabolic indicators might suggest a crosstalk between them. CRP could be the intermediate factor between these indicators and chronic diseases such as hypertension, diabetes mellitus, and cardiovascular diseases as well as obesity. Evidences supporting this hypothesis could also be found in previous studies. In a study on the relationship between obesity and CRP in the children, the results showed that the obese children had significantly increased CRP. In addition, leptin and insulin also significantly increased comparing to the control group. In this study, BMI had positive correlation with CRP and negative correlation with insulin sensitive index. Leptin had positive correlation between insulin and BMI while it had negative correlation with insulin sensitive index. These suggested that obese children may have insulin resistance and leptin resistance. Both CRP and leptin could play important role in the process of obesity. In overweight and obese adults, elevated level of CRP was also seen [26, 27]. In this study, higher BMI was associated with higher CRP concentrations, suggesting a state of low-grade systemic inflammation in overweight and obese adults. In the obese persons, expanded adipose tissue secreted proinflammatory cytokines which enhance hepatic synthesis of CRP. CRP could bind to LDL, VLDL, and oxidized LDL and promote complement activation. The interaction of CRP and LDL might further affect lipid metabolism and promote obese and other chronic diseases. Studies also supported that CRP had direct proinflammatory effect and was an independent risk factor in a variety of cardiovascular diseases and might be used as a biomarker to predicate the risk of cardiovascular disease and to predicate the long-term outcome of acute coronary syndrome [28–30]. Therefore, CRP could function as an inflammatory marker and mediated the function of lipid protein in obese and cardiovascular disease. Correlation studies on obesity and type II diabetes also showed that significantly high level of CRP was seen in obese people both in diabetes group and control group, suggesting that CRP might be the major link between obesity and type II diabetes. Therefore, integrated health management intervention including both dietary supplements and health managements might affect metabolic indicators directly or indirectly by CRP to control obesity. CRP level might be used as a promising marker to assess improvements in obesity.

## Figures and Tables

**Table 1:** Sample sizes and loss to follow-up ratios at different stages.

Intervention time	Total	
	*N*	Loss of follow-up (%)
Baseline	1488	—
1 month	1420	4.57
3 months	1380	7.26
6 months	1271	14.58
9 months	1186	20.3
12 months	1092	26.61

**Table 2:** Effects of health interventions to physical indicators and correlation between dietary supplements and health management.

Group	*N*	Bodyweight		BMI		BFR		Waistline		Hipline		Annual weight loss of 5%	
		Baseline	Annual reduction	Baseline	Annual reduction	Baseline	Annual reduction	Baseline	Annual reduction	Baseline	Annual reduction	Effective population	%
P + GM	271	77.51 ± 13.35	−0.23 ± 2.70	28.67 ± 3.47	0.06 ± 1.07	34.72 ± 7.59	0.64 ± 2.79	94.20 ± 9.04	1.97 ± 4.62	101.24 ± 6.81	0.46 ± 2.78	16	5.9
P + HM	270	79.18 ± 13.69	1.98 ± 3.73	29.23 ± 3.52	0.85 ± 1.40	35.94 ± 7.56	2.13 ± 3.09	94.38 ± 9.93	3.58 ± 4.52	102.51 ± 7	1.94 ± 3.24	72	26.67
DS + GM	279	79.27 ± 14.17	3.15 ± 3.90	29.19 ± 3.72	1.24 ± 1.41	36.22 ± 7.53	2.49 ± 3.20	95.52 ± 10.42	5.44 ± 4.97	102.21 ± 7.49	2.57 ± 3.53	100	35.84
DS + HM	272	79.02 ± 13.81	4.24 ± 4.13	29.30 ± 3.87	1.60 ± 1.80	36.01 ± 7.82	3.04 ± 3.29	95.50 ± 9.96	6.61 ± 4.96	102.25 ± 6.86	3.25 ± 3.33	119	43.75
Variance analysis of factorial design													
DS		<0.001		<0.001		<0.001		<0.001		<0.001		Chi-square value: 107.58 *p* value: <0.0014	
HM		<0.001		<0.001		<0.001		<0.001		<0.001			
DS *∗* HM		0.012		0.013		0.013		0.444		0.034			

*Note*. Measured data is expressed in mean ± standard deviation. The statistical methods include factorial design variance analysis, single-factor variance analysis, and chi-square test, where *p* < 0.05 denotes a statistically significant difference. P: placebo; DS: dietary supplements; GM: general management; H: health management.

DS *∗* HM: cross function of dietary supplements and health management.

**Table 3:** Annual change of metabolic indicators after one-year intervention.

		*N*	Baseline	12 months	Annual decline	*p*
Lnhs-CRP	P + GM	270	0.05 ± 1.07	0.28 ± 1.04	−0.24 ± 0.95	<0.001
	P + HM	268	0.03 ± 1.17	0.24 ± 1.17	−0.15 ± 0.96	0.011
	DS + GM	277	0.02 ± 1.11	0.15 ± 1.04	−0.15 ± 0.79	0.002
	DS + HM	271	0.05 ± 1.16	0.07 ± 1.1	0 ± 0.98	0.999
TC	P + GM	270	4.44 ± 0.87	4.59 ± 0.91	−0.15 ± 0.73	0.001
	P + HM	268	4.42 ± 0.83	4.55 ± 0.85	−0.14 ± 0.73	0.002
	DS + GM	277	4.52 ± 0.88	4.58 ± 0.88	−0.06 ± 0.67	0.151
	DS + HM	271	4.5 ± 0.88	4.67 ± 0.91	−0.17 ± 0.67	<0.001
TG	P + GM	268	1.16 ± 1	1.76 ± 1.31	−0.14 ± 0.91	0.011
	P + HM	267	1.64 ± 1.2	1.57 ± 1.17	0.04 ± 0.75	0.372
	DS + GM	277	1.57 ± 1.04	1.41 ± 0.88	0.16 ± 0.82	0.001
	DS + HM	270	1.55 ± 1.03	1.33 ± 0.9	0.22 ± 0.93	<0.001
HDL-c	P + GM	270	1.31 ± 0.29	1.29 ± 0.24	−0.02 ± 0.21	0.204
	P + HM	268	1.31 ± 0.28	1.32 ± 0.27	0.01 ± 0.23	0.677
	DS + GM	277	1.35 ± 0.26	1.32 ± 0.25	−0.03 ± 0.23	0.042
	DS + HM	271	1.32 ± 0.26	1.36 ± 0.29	0.05 ± 0.25	0.001
LDL-c	P + GM	270	2.91 ± 0.73	3.07 ± 0.85	−0.03 ± 0.68	0.523
	P + HM	268	2.88 ± 0.82	2.98 ± 0.88	0.03 ± 0.77	0.475
	DS + GM	277	2.97 ± 0.78	3.15 ± 0.89	−0.01 ± 0.64	0.881
	DS + HM	271	2.96 ± 0.8	3.23 ± 0.92	−0.1 ± 0.68	0.017
HbA1C%	P + GM	271	5.68 ± 0.68	5.59 ± 0.8	0.24 ± 0.49	<0.001
	P + HM	270	5.63 ± 0.66	5.52 ± 0.77	0.29 ± 0.7	<0.001
	DS + GM	278	5.64 ± 0.69	5.49 ± 0.6	0.32 ± 0.65	<0.001
	DS + HM	272	5.56 ± 0.69	5.49 ± 0.62	0.31 ± 0.55	<0.001
LnLEP	P + GM	268	3.15 ± 0.83	3.13 ± 0.85	−0.02 ± 0.47	0.55
	P + HM	269	3.13 ± 0.94	3.16 ± 0.85	0.03 ± 0.41	0.298
	DS + GM	275	3.16 ± 0.84	3.12 ± 0.85	0.08 ± 0.44	0.003
	DS + HM	271	3.16 ± 0.92	2.99 ± 0.92	0.16 ± 0.4	<0.001
LnINS	P + GM	271	2.32 ± 0.51	2.48 ± 0.67	−0.16 ± 0.66	<0.001
	P + HM	265	2.36 ± 0.49	2.33 ± 0.61	0.04 ± 0.58	0.259
	DS + GM	277	2.34 ± 0.5	2.18 ± 0.59	0.16 ± 0.55	<0.001
	DS + HM	271	2.45 ± 0.52	2.16 ± 0.63	0.29 ± 0.62	<0.001
GLU	P + GM	270	5.03 ± 1.01	5.27 ± 1.5	−0.16 ± 0.94	0.006
	P + HM	268	4.95 ± 1.22	5.05 ± 1.24	−0.06 ± 1.05	0.388
	DS + GM	277	5.02 ± 1.25	4.97 ± 1.12	0.16 ± 0.82	0.001
	DS + HM	271	4.94 ± 1.07	4.93 ± 0.99	0.03 ± 0.84	0.583
HOMAIR	P + GM	268	2.39 ± 1.73	3.59 ± 3.75	−0.96 ± 3.67	<0.001
	P + HM	265	2.3 ± 1.81	2.86 ± 2.7	0.64 ± 2.24	0.435
	DS + GM	275	2.48 ± 2.35	2.38 ± 2.15	0.32 ± 2.16	0.013
	DS + HM	270	2.36 ± 2.23	2.35 ± 2.17	0.64 ± 2.24	<0.001
HOMAIS	P + GM	268	0.03 ± 0.02	0.02 ± 0.01	−0.0022 ± 0.01	0.008
	P + HM	269	0.03 ± 0.02	0.02 ± 0.02	0.002 ± 0.02	0.037
	DS + GM	275	0.03 ± 0.02	0.03 ± 0.02	0.0049 ± 0.02	<0.001
	DS + HM	271	0.03 ± 0.02	0.03 ± 0.02	0.0085 ± 0.02	<0.001
LnTNF	P + GM	268	3 ± 0.98	3.15 ± 0.83	−0.14 ± 0.73	0.001
	P + HM	269	2.99 ± 1.04	3.01 ± 0.99	−0.04 ± 0.8	0.406
	DS + GM	275	2.93 ± 1.04	3.02 ± 0.98	−0.16 ± 0.82	0.001
	DS + HM	271	2.97 ± 0.95	3.03 ± 0.92	−0.12 ± 0.75	0.006
FFA	P + GM	271	0.47 ± 0.22	0.5 ± 0.26	0.02 ± 0.32	0.215
	P + HM	269	0.48 ± 0.19	0.51 ± 0.3	0 ± 0.33	0.901
	DS + GM	276	0.48 ± 0.21	0.58 ± 0.27	−0.04 ± 0.34	0.071
	DS + HM	269	0.45 ± 0.21	0.56 ± 0.31	0 ± 0.36	0.966
LnADI	P + GM	268	7.71 ± 0.63	7.66 ± 0.8	0.01 ± 0.55	0.794
	P + HM	269	7.73 ± 0.66	7.75 ± 0.71	0.03 ± 0.46	0.22
	DS + GM	275	7.71 ± 0.67	7.74 ± 0.69	0.06 ± 0.46	0.044
	DS + HM	270	7.75 ± 0.77	7.77 ± 0.75	0.03 ± 0.48	0.378

*Note*. P: placebo; DS: dietary supplements; GM: general management, H: health management.

DS *∗* HM: cross function of dietary supplements and health management.

**Table 4:** Factorial and interaction analysis of metabolic factors.

	Mixed linear model	*F*	*p*
Lnhs-CRP	DS	4.14	0.017
	HM	2.168	0.115
	DS *∗* HM	0.31	0.734
LnTNF	DS	0.764	0.466
	HM	1.733	0.177
	DS *∗* HM	0.579	0.561
LnADI	DS	1.423	0.241
	HM	0.828	0.437
	DS *∗* HM	0.638	0.505
LnLEP	DS	9.676	0
	HM	4.058	0.017
	DS *∗* HM	1.191	0.304
LnINS	DS	35.824	0
	HM	10.349	0
	DS *∗* HM	2.606	0.074
TC	DS	0.363	0.696
	HM	2.226	0.108
	DS *∗* HM	1.048	0.351
TG	DS	13.974	0
	HM	4.401	0.012
	DS *∗* HM	1.445	0.236
HDL-c	DS	1.738	0.176
	HM	6.567	0.001
	DS *∗* HM	2.609	0.074
LDL-c	DS	3.31	0.037
	HM	0.459	0.632
	DS *∗* HM	2.395	0.092
GLU	DS	9.095	0
	HM	0.738	0.478
	DS *∗* HM	2.455	0.086
FFA	DS	5.746	0.003
	HM	0.833	0.435
	DS *∗* HM	1.115	0.328
HbA1C%	DS	1.349	0.26
	HM	1.319	0.268
	DS *∗* HM	1.21	0.298
HOMAIR	DS	25.728	0
	HM	6.568	0.001
	DS *∗* HM	2.836	0.059
HOMAIS	DS	28.597	0
	HM	7.57	0.001
	DS *∗* HM	0.064	0.938

*Note*. Single-factor variance analysis was used, where *p* < 0.05 denotes a statistically significant difference.

P: placebo; DS: dietary supplements; GM: general management, H: health management.

DS *∗* HM: cross function of dietary supplements and health management.

**Table 5:** Correlation between weight loss and metabolic indicators and correlation factor.

* *	Over all	
	*R*	*p*
Decline in hs-CRP	0.234	<0.001
Decline in TC	0.11	<0.001
Decline in TG	0.285	<0.001
Rise in HDL	0.082	0.007
Decline in LDL	0.093	0.002
Decline in GLU	0.134	<0.001
Decline in HbA1C	0.1	0.001
Decline in LnINS	0.343	<0.001
Decline in LnLEP	0.096	0.002
Rise in HOMAIR	0.258	0.002
Rise in HOMAIS	0.295	<0.001
Rise in LnADI	0.02	0.514
Decline in FFA	−0.012	0.687
Decline in LnTNF	0.027	0.382

*Note*. Multiple logistic regression analysis was used for testing the correlation between weight loss and metabolic indicators and correlation factor.
